# Understanding the host expansion and interspecies infection of peste des petits ruminants virus in view of global control and eradication

**DOI:** 10.3389/fcimb.2026.1777301

**Published:** 2026-02-23

**Authors:** Mousumi Bora, Monu Karki

**Affiliations:** Department of Veterinary Microbiology, College of Veterinary Science, Guru Angad Dev Veterinary and Animal Sciences University, Ludhiana, Punjab, India

**Keywords:** global eradication, host range, interspecies transmission, PPR virus, SLAM (CD150), virus evolution

## 1 Introduction

Peste-des-petits ruminants (PPR), also known as plague of sheep and goat is an impactful barrier to small ruminant farming (Munir, 2014). This highly contagious disease threatens more than 2.1 billion small ruminant population in Asia and Africa that provide livelihood and food security to underprivileged sections of marginalized rural communities (FAO and WOAH, 2025). PPR is caused by the peste-des petits-ruminants virus (PPRV), a virus belonging to the genus Morbillivirus under the family Paramyxoviridae (ICTV, 2025). Due to its significant economic impact in developing nations and rural economies, the Food and Agricultural Organization of the United Nations (FAO) and World Organization for Animal Health (WOAH) jointly initiated Peste-des-petits ruminants Global Eradication Programme (PPR-GEP) in 2015 with a mandate to eradicate the disease by 2030 (FAO OIE, 2015).

Although sheep and goats are the primary target hosts of the PPRV, an increasing number of reports have documented sporadic infections and associated mortality among wildlife species across endemic regions (SowjanyaKumari et al., 2021).Clinical and laboratory confirmation of PPRV together with serological testimony in numerous wildlife hosts (FAO and OIE., 2015; SowjanyaKumari et al., 2021) suggests that PPR is an emerging disease in wild caprines, wild ungulates and camels (Rahman et al., 2020; Fine et al., 2020). Despite clinical, serological, virological and genomic evidence implicating PPRV in disease manifestations among wildlife hosts, the epidemiology of the virus in these species remains poorly understood and continues to pose a complex scientific challenge. What precise role the newly accrued list of hosts plays in the emergence, transmission and maintenance of PPRV in nature remains elusive. The potential impact of a broad range of susceptible wildlife hosts coexisting in close proximity to domestic sheep and goats on the transmission dynamics and success of PPR control and eradication initiatives requires critical and comprehensive evaluation.

Prior to the PPR-GEP, the Global Rinderpest Eradication Programme (GREP) revealed that rinderpest was eliminated from occasionally susceptible wildlife once transmission was controlled in its natural hosts (cattle and buffalo) through extensive vaccination, thereby creating a strong barrier to spillover (Morens et al., 2011). However, global PPR eradication efforts are unlikely to follow the same trajectory as rinderpest eradication due to fundamental differences in host dynamics, viral characteristics and ecological complexity. Small ruminants exhibit high population turnover, genetic diversity and mobility, in contrast to large ruminants (Akinmoladun et al., 2019). Moreover, PPRV exists as four genetically distinct lineages (I–IV), all capable of causing disease and rapid geographic spread (Albina et al., 2013). Control of PPR in wildlife is therefore critical. Consequently, a comprehensive understanding of host susceptibility and transmission dynamics is essential for implementing an effective PPR-GEP.

## 2 Drivers of host expansion and interspecies transmission

Peste-des petits-ruminants virus (PPRV), traditionally confined to domestic small ruminants like sheep and goats, has demonstrated a notable expansion in host range and inter-species transmission (**Table 1**). The host expansion of PPRV is the outcome of combination of its genetic adaptability, receptor conservation and increased ecological interfaces between domestic and wild species (Jones et al., 2021; Barman et al., 2024; Wei et al., 2025). These factors together increase the virus’s ability to infect, adapt and persist in atypical hosts providing opportunities for viral adaptation and mutation (Ul-Rahman et al., 2022). Understanding the expanding host range of PPRV is crucial, as it provides insights into the virus’s potential to cause disease across diverse species which may pose future challenges to PPR-GEP. Increasing evidence indicates that the virus occurs in a wide range of other domestic and wild animal species, including cattle, buffaloes, camels, cervids and even water buffaloes, with or without the manifestation of clinical signs (Aziz-Ul-Rahman et al., 2018; Ul-Rahman et al., 2022). The host range expansion of PPRV has been continuous, with reports ranging from infection in endangered Mongolian saiga antelopes (Pruvot et al., 2020) to documented exposure in pigs (Adedeji et al., 2025).

**Table 1:** Rising reports of Peste-des-petits ruminants (PPR) infection in wildlife hosts/atypical hosts.

Common name	Scientific name	Type of sample	Detection method	Country reported	References
Afghan markhor	Capra falconeri	Tissue and sera	Immunohistochemistry, c-ELISA and RT-PCR	UAE	Kinne et al., 2010
Arabian gazelle	Gazella gazelle	Tissue	Immunohistochemistry, c-ELISA and RT-PCR	UAE	Kinne et al., 2010
		Oculo-nasal swab and sera	c-ELISA, RT-PCR and isolation	Saudi Arabia	Sharawi et al., 2010
Barbary sheep	Ammotragus lervia	Tissue and sera	Immunohistochemistry, c-ELISA and RT-PCR	UAE	Kinne et al., 2010
Barking deer	Muntiacus muntjak	Tissue	RT-PCR	India	Barman et al., 2024
Bharal	Pseudois nayaur	Tissue and sera	c-ELISA and RT-PCR	China	Bao et al., 2011
Bubal hartebeests	Alcelaphus boselaphus	Nasal swabs and sera	c-ELISA and RT-PCR	Ivory Coast	Couacy-Hymann et al., 2005
Buffaloes	Syncerus caffer	Sera	c-ELISA and RT-qPCR	Ivory Coast; Tanzania; Uganda	Couacy-Hymann et al., 2005
		Sera	c-ELISA	Uganda	Fernandez Aguilar et al., 2020
		Sera	c-ELISA	Tanzania	Mahapatra et al., 2015
		Sera	c-ELISA	Indonesia	Sendow et al., 2024
Bushbucks	Tragelaphus scriptus	Tissue and sera	Immunohistochemistry, c-ELISA and RT-PCR	UAE	Kinne et al., 2010
Camel	Camelus dromedarius	Sera	c-ELISA	Ethiopia	Roger et al., 2001
		Tissue	immunocapture ELISA and RT-PCR	Sudan	Kwiatek et al., 2011
		Tissue	AGDT, immune capture ELISA, RT-PCR and virus isolation	Sudan	Khalafalla et al., 2010
Cattle	Bos taurus	Sera	c-ELISA	India	Balamurugan et al., 2014
		Sera	c-ELISA	Nepal	Prajapati et al., 2021
Chowsingha/Four-horned antelope	Tetracerus quadricornis	Tissue	RT-PCR	India	Jaisree et al., 2018
		Tissue	RT-PCR	India	Barman et al., 2024
Defassa waterbuck	Kobus defassa	Nasal swabs and sera	c-ELISA and RT-PCR	Ivory Coast	Couacy-Hymann et al., 2005
Dorcas gazelles	Gazella dorcas	Sera and tissue	AGID, VNT, FAT and virus isolation	Saudi Arabia	Elzein et al., 2004
		Sera	c-ELISA	Nigeria	Bello et al., 2016
		Ocular and nasal swabs, sera and tissue	immune capture ELISA and RT-PCR	Sudan	Asil et al., 2019
Goitered gazelle	Gazella subgutturosa	Sera	c-ELISA and VNT	Turkey	Gür and Albayrak, 2010
Grant’s gazelle	Nanger granti	Sera; Eye and nasal swabs	c-ELISA and RT-qPCR	Tanzania	Mahapatra et al., 2015
Grey duiker	Sylvicapra grimmia	Sera	c-ELISA	Nigeria	Ogunsanmi et al., 2003
Hog Deer	Axis porcinus	Tissue	RT-PCR	India	Barman et al., 2024
Ibex	Capra ibex	Tissue	RT-PCR and virus isolation	China	Zhu et al., 2016
Impala	Aepyceros melampus	Sera and tissue	Immunohistochemistry, c-ELISA and RT-PCR	UAE	Kinne et al., 2010
		Sera	c-ELISA	Tanzania	Mahapatra et al., 2015
Indian Buffalo	Bubalus bubalis	Tissue	HI, RT-PCR and virus isolation	India	Govindarajan et al., 1997
Kobs	Kobus kob	Sera	c-ELISA	Ivory Coast;	Couacy-Hymann et al., 2005
		Sera	c-ELISA	Uganda	Fernandez Aguilar et al., 2020
Mongolian antelope	Saiga tatarica mongolica	Eye swabs, tissue, faeces and saliva	LFD and RT-PCR	Mongolia	Pruvot et al., 2020
Mouse deer	Moschiola indica	Tissue	RT-PCR	India	Barman et al., 2024
Pigs	Sus scrofa domesticus	Sera	c-ELISA	Nigeria	Adedeji et al., 2025
Sindh ibex	Capra aegagrus blythi	Tissue	RT-PCR	Pakistan	Abubakar et al., 2011
Springbuck	Antidorcas marsupialis	Sera and tissue	Immunohistochemistry, c-ELISA and RT-PCR	UAE	Kinne et al., 2010
Thamin	Rucervus eldii	Tissue	RT-PCR	India	Barman et al., 2024
Thompson’s gazelle	Eudorcas thomsonii	Tissue	VNT and virus isolation	Saudi Arabia	Elzein et al., 2004
Water Deer	Hydropotes inermis	Tissue	Electron microscopy, RT-PCR and virus isolation	China	Zhou et al., 2018
Wild goat	Capra aegagrus	Sera and tissue	PPR blocking ELISA and RT-qPCR	Kurdistan	Hoffmann et al., 2012
Wildebeest	Connochaetes gnou	Sera	c-ELISA	Tanzania	Mahapatra et al., 2015
Yak	Bos grunniens	Sera	c-ELISA	Pakistan	Abubakar et al., 2019
		Sera	c-ELISA	Tajikistan	Abubakar et al., 2019

The mechanisms underlying this host expansion could be multifaceted. One of the factors contributing to inter-species transmission of PPRV is predicted to be the variation in the cellular receptors across species, influencing the virus’s ability to infect different hosts (Wei et al., 2025). Signaling lymphocyte activation molecule (SLAM/CD150) and poliovirus receptor-like 4 (Nectin-4/PVRL4) serve as the primary cellular receptors binding the heamaglutinin (H) protein facilitating entry of PPRV, into the host cells (Prajapati et al., 2019). These receptors are highly conserved among different mammalian species, particularly within Artiodactyla (even-toed ungulates), enabling the virus to attach and infect non-traditional hosts (Jones et al., 2021). Supporting this, comparative analyses of the SLAM receptor across PPRV-susceptible species have identified 14 least common amino acid sites, and based on these conserved motifs, as many as 48 species across 20 families have been predicted to be potentially susceptible to PPRV infection (Fan et al., 2024). Furthermore, evolutionary studies of the PPRV H protein across diverse isolates suggest co-evolution with host cellular receptors, highlighting their role in shaping host specificity and cross-species transmission potential (Dou et al., 2020). Consistent with these studies, interaction energy and surface contact analyses between PPRV-H and SLAM proteins from different species have demonstrated the strongest binding affinity with sheep SLAM, followed by receptors from other susceptible species, underscoring the relevance of receptor-virus compatibility in determining host susceptibility (Liang et al., 2016). Furthermore, climate variability and landscape change can indirectly modulate PPRV transmission by altering host distribution, movement patterns and wildlife-livestock interfaces (Balamurugan et al., 2021; Zeng et al., 2021; Nkamwesiga et al., 2022; Chai et al., 2026). Changes in temperature, rainfall patterns and grazing landscapes can enhance animal aggregation and cross-species contact, facilitating spillover of PPRV from domestic small ruminants to wildlife (Mahapatra et al., 2015; Jones et al., 2021).

## 3 Global efforts for control and eradication

The economic impact of PPR outbreaks is extremely high, with the adverse effects disproportionately affecting socio-economically underprivileged communities, reflecting their intrinsic dependence on sheep and goat farming (Jones et al., 2016). PPR free farming would confer substantial direct benefits by safeguarding the livelihoods of millions of livestock farmers, predominantly in developing countries, and could potentially uplift rural economies above the poverty threshold. Recognizing the severe global economic losses associated with PPR, the FAO and the WOAH launched PPR Global Control and Eradication Strategy (PPR-GCES) in 2015, aimed at achieving a PPR-free world by 2030 through three interrelated components: (i) control and eradication of PPR, (ii) strengthening of veterinary services, and (iii) prevention and control of other major diseases affecting small ruminants (FAO and OIE, 2015). The undiscounted costs of implementing the 15-year global strategy are estimated to range between US$ 7.6 and 9.1 billion, with the initial five-year phase requiring an investment of approximately US$ 2.5-3.1 billion (FAO and OIE, 2015). Vaccination campaigns are expected to play a pivotal role across all scenarios, as intensive and targeted immunization of at-risk populations guided by robust epidemiological and economic analyses could substantially reduce overall costs. Overall, the annual costs during the initial five-year period are projected to be approximately US$ 0.5 billion (FAO and OIE, 2015). Currently, the direct economic losses attributed to PPR are estimated at US$ 1.2-1.7 billion annually. Successful implementation of PPR-GEP would eliminate these losses entirely, underscoring the substantial economic and social returns of investing in PPR eradication.

The rising number of PPRV reports in wildlife species underscores their increasingly important role in PPR epidemiology. Since 2010, the number of reporting years has approximately doubled, accompanied by a more than twofold expansion in the diversity of affected wildlife species. The following control strategies targeting wild and atypical hosts could play a critical role in interrupting virus transmission cycles and substantially advancing global eradication efforts:

### 3.1 Sentinel surveillance

Current evidence indicates that PPRV detection in wildlife and large ruminants primarily reflects spillover from infected sheep and goats (Kinne et al., 2010; Abubakar et al., 2011; Mahapatra et al., 2015; Barman et al., 2024). Such spillover events can be exploited for sentinel surveillance using robust serological diagnostics targeting PPRV specific antibodies. Because PPR in small ruminants may remain subclinical or mild, surveillance in susceptible wildlife and atypical hosts can help reveal silent PPRV circulation. Reports of PPRV seropositivity in cattle, buffalo and pigs, despite absence of clinical disease, support the use of large ruminants as sentinel indicators of asymptomatic transmission, particularly in vaccinated or low incidence settings (Agga et al., 2019).

### 3.2 Targeted wildlife health surveillance

In view of increasing evidence of PPRV infection in wildlife, diagnostic measures targeting wildlife populations are now under consideration for integration into successive phases of the PPR-GEP (Fine et al., 2020). Diagnostic testing of wildlife samples should comply with the WOAH Terrestrial Manual for PPR, currently validated for sheep and goats (OIE and FAO, 2021). Active PPRV infection is confirmed by molecular detection of viral RNA, typically by RT-PCR, using nasal, ocular or oral swabs from captive wildlife or tissues collected from carcasses during mortality events. Serological surveillance to detect prior exposure relies on competitive ELISA (c-ELISA) or virus neutralization tests (VNT) (WOAH Terrestrial Manual, 2022) and generally requires blood sampling, most feasible during opportunistic capture, translocation or wildlife management interventions. Given the logistical, financial and ethical challenges of wildlife capture, the development of non-invasive diagnostic tools should be prioritized for surveillance studies (OIE and FAO, 2021). However, owing to the logistical, financial and ethical challenges associated with wildlife capture, non-invasive diagnostic approaches should be prioritized (OIE and FAO, 2021). In this context, faecal sampling has gained increasing attention as a viable alternative to blood collection for PPRV surveillance in wildlife (Bataille et al., 2019; Halecker et al., 2020).

### 3.3 Vaccination

Evidence indicates that camels and wild small ruminant species are vulnerable to PPRV infection (Roger et al., 2001; Khalafalla et al., 2010). Vaccination of susceptible wildlife and unusual host species in protected settings such as zoos, national parks and wildlife reserves could be beneficial, particularly for endangered species or captive breeding programmes aimed at conservation. In addition, creating strong herd immunity in domestic small ruminants at wildlife-livestock interfaces could help prevent spillover transmission (Cameron, 2019).

### 3.4 Quarantine and restricted animal movements

Given the limited trade volume of wildlife and unusual hosts, enforcing strict quarantine at trade points and prior to introduction into new habitats is feasible and essential. It is recommended that animals imported from countries or zones considered infected with PPRV be maintained under quarantine for at least 21 days at a designated quarantine station to prevent potential disease introduction (WOAH, 2021). Restricting animal movements at grazing and drinking points can further reduce wildlife-livestock interactions and interrupt sylvatic-domestic transmission cycles (Jones et al., 2021).
